# Microbial Functional Potentials Differ Among Monospecific and Mixed Moss Biocrusts in an Alpine Sandy Ecosystem

**DOI:** 10.3390/biology15161372

**Published:** 2026-08-12

**Authors:** Meiling Liu, Zhihui Wang, Ruiqing Zhu, Huichun Xie, Kunyuan Wanghe

**Affiliations:** 1 Key Laboratory of Tibetan Plateau Land Surface Processes and Ecological Conservation (Ministry of Education), College of Life Sciences, Qinghai Normal University, Xining 810016, China; liuml@lzb.ac.cn (M.L.); 13109719325@163.com (Z.W.); yezino.1@163.com (H.X.); 2 Academy of Plateau Science and Sustainability, Qinghai Normal University, Xining 810016, China; 3 Qilian Mountain Southern Slope Forest Ecosystem Research Station, Haidong 810700, China; 4 Northwest Institute of Plateau Biology, Chinese Academy of Sciences, Xining 810016, China; wanghekunyuan@189.cn

**Keywords:** biological soil crusts, moss crust category, alpine sandy ecosystem, functional potential, CAZy, carbon cycling, nitrogen cycling, mixed-crust midpoint deviation

## Abstract

Moss-dominated biological soil crusts are associated with carbon and nitrogen cycling in dryland and alpine sandy ecosystems, but functional differentiation among closely related moss species and their mixed assemblages remains unclear. We used shotgun metagenomic sequencing to compare below-crust soil associated with *Didymodon constrictus* (Mitt.) K. Saito (*D. constrictus*) crusts (mossC), *ferrugineus* (Schimp. ex Besch.) M.O. Hill (*D. ferrugineus*) crusts (mossF), and visually co-dominated mixed crusts (mossM) in the Gonghe Basin on the northeastern Qinghai–Tibet Plateau. Fifteen spatially separated quadrats per category were pooled into three composite biological replicates (effective *n* = 3). KEGG, CAZy, and targeted carbon- and nitrogen-cycling annotations showed category-associated differences in relative gene representation. MossC had greater mean representation of glycoside hydrolases and several complex-carbon-processing functions, mossF had greater representation of nitrogen-assimilation and acetate-related functions, and mossM had greater representation of selected carbon-degradation, nitrogen-mineralization, and dissimilatory-nitrate-reduction functions. The full RDA model explained 58.4% of functional variation (adjusted R^2^ = 0.334; exact permutation *p* = 0.028340), with single-variable associations retained for total carbon and soil water content. Genus-level taxonomic dissimilarity correlated with KEGG, CAZy, carbon-cycling, and nitrogen-cycling dissimilarities after FDR correction. MossM showed both positive and negative descriptive deviations from the approximate unweighted midpoint of mossC and mossF, but no inferential test was applied to these deviations. The small number of composite replicates, visually estimated moss proportions, edaphic confounding, and absence of activity measurements limit causal and confirmatory interpretation.

## Simple Summary

Biological soil crusts are thin living layers at the soil surface that contribute to soil stability and nutrient cycling. We compared below-crust microbial gene profiles associated with two monospecific moss crusts and a visually co-dominated mixed crust in an alpine sandy ecosystem. The effective sample size was three composite biological replicates per category. The crust categories differed in the relative representation of genes associated with carbohydrate processing and carbon and nitrogen transformations. The mixed-crust mean also showed positive and negative descriptive deviations from the approximate unweighted midpoint of the two monospecific means. Because moss proportions were visually estimated, soil properties differed among categories, and process rates were not measured, these patterns represent site-specific metagenomic functional potential rather than confirmed effects on ecosystem activity.

## 1. Introduction

Biological soil crusts (biocrusts) are communities of cyanobacteria, algae, fungi, lichens, bryophytes, and associated heterotrophic microorganisms that organize the soil surface and contribute to erosion control, water redistribution, and carbon and nitrogen cycling [1,2,3,4]. Moss-dominated biocrusts generally represent a structurally developed state with greater surface roughness, biomass, and water-retention capacity than early cyanobacterial crusts [5,6]. Their associated microbial communities remain insufficiently resolved at the level of functional gene repertoires, particularly in cold, wind-eroded alpine sandy ecosystems.

Mosses are non-vascular, poikilohydric plants whose shoot water content responds rapidly to atmospheric and substrate moisture. Many dryland mosses suspend metabolism during dehydration and resume physiological activity after rehydration, although recovery varies among taxa and environmental conditions [5,7]. *Didymodon constrictus* (Mitt.) K. Saito (*D. constrictus*) and *D. ferrugineus* (Schimp. ex Besch.) M.O. Hill (*D. constrictus*) are acrocarpous mosses of the family Pottiaceae. *D. constrictus* is a small- to medium-sized moss, approximately 10–25 mm tall, with yellowish-green to reddish-brown shoots forming loose or dense tufts on exposed soil and rocky substrates [8]. *D. ferrugineus* generally forms loose yellowish-green to reddish-brown tufts, spreading to recurved leaves when moist, and grows on soil, ledges, rocky outcrops, and calcareous substrates, including alpine and tundra habitats [8,9]. Both species occur in alpine grasslands of the Qinghai–Tibet Plateau [10].

Unlike vascular plants, mosses lack true roots and do not form a conventional rhizosphere through root exudation. Their rhizoids nevertheless create a close soil-contact interface, while soluble compounds released during wetting–drying cycles, senescent tissues, and particles trapped within the turf can supply microbial substrates and modify microsite moisture and temperature [11,12,13]. Moss growth form, shoot density, tissue chemistry, and water retention could therefore be associated with distinct below-crust microbial communities. However, these species-level mechanisms were not measured here and are treated as ecological hypotheses rather than demonstrated traits.

The Gonghe Basin on the northeastern Qinghai–Tibet Plateau was selected because it is an extensive alpine sandy ecosystem affected by low temperatures, limited precipitation, high evaporation, strong solar radiation, and frequent wind erosion. Approximately one-third of the basin has experienced desertification [14], creating sandy surfaces with low water retention and resource availability. Such alpine sandy ecosystems are commonly nutrient-limited, particularly with respect to nitrogen, because sparse vegetation restricts organic inputs, while low temperatures, limited soil moisture, and coarse-textured soils constrain nitrogen accumulation and transformation [12,14]. Under these conditions, biocrust-associated microorganisms mediate nitrogen fixation, organic nitrogen mineralization, assimilation, nitrification, denitrification, and dissimilatory nitrate reduction to ammonium, while soluble nutrients released from biocrusts can also be transferred to the underlying mineral soil [2,3,4,11]. The balance between nitrogen-retaining processes, such as microbial assimilation and dissimilatory nitrate reduction to ammonium, and potential nitrogen-loss pathways, such as denitrification, is therefore important for maintaining soil fertility and supporting ecosystem development [3,4,12]. Comparing nitrogen-cycling functional genes among monospecific and mixed moss biocrusts can thus clarify whether moss identity and coexistence are associated with different capacities for nitrogen acquisition, retention, and potential loss in this nutrient-limited alpine sandy ecosystem.

Mixed moss crusts may differ from either monospecific crust because coexistence can increase spatial heterogeneity in detrital inputs, hydration, and redox conditions. Biodiversity–ecosystem-function theory predicts that mixtures may show selective departures from the midpoint of their component systems [15]. Shotgun metagenomics characterizes community gene repertoires and therefore supports inference about functional potential, not gene expression or process rates. KEGG provides orthology and pathway assignments, whereas CAZy/dbCAN2 and NCycDB support targeted annotation of carbohydrate-active enzymes and nitrogen-cycling genes [16,17,18]. We compared below-crust microbial functional profiles associated with *D. constrictus* crusts (mossC), *D. ferrugineus* crusts (mossF), and visually co-dominated mixed crusts (mossM). We aimed to (i) characterize overall functional gene profiles; (ii) compare KEGG functions, carbohydrate-active enzymes, and carbon- and nitrogen-cycling genes among crust categories; and (iii) describe whether the mossM group mean departed from the approximate unweighted midpoint of the two monospecific group means. All midpoint departures were treated as exploratory descriptive patterns.

## 2. Materials and Methods

### 2.1. Study Site and Sample Collection

**Figure 1:**
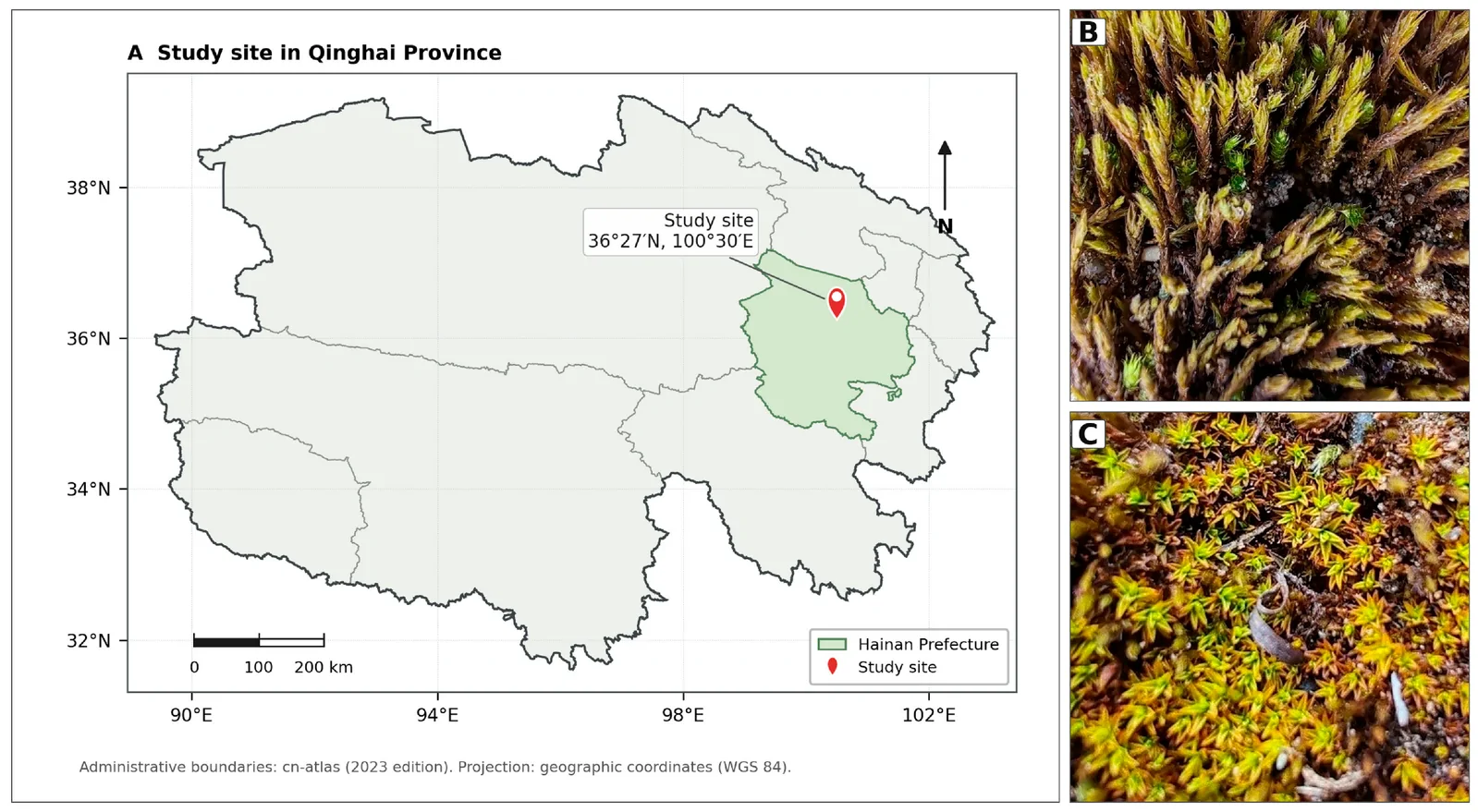
Location of the study site and representative photographs of the two monospecific moss biocrusts. (**A**) Study site in Qinghai Province, China (36°27′ N, 100°30′ E). Administrative boundaries are based on the cn-atlas 2023 edition and geographic coordinates use WGS 84. (**B**) *Didymodon constrictus* (Mitt.) K. Saito (*D. constrictus*) biocrust. (**C**) *D. ferrugineus* (Schimp. ex Besch.) M.O. Hill (*D. constrictus*) biocrust. No photograph of the visually mixed mossM category was available.

Sampling was conducted at the Shazhuyu Experimental Station in the Gonghe Basin, Qinghai Province, China (36°27′ N, 100°30′ E; Figure 1). Three moss-crust categories were identified: *D. constrictus* crust (mossC), *D. ferrugineus* crust (mossF), and mixed crust visually co-dominated by both species (mossM). For each category, fifteen 10 cm × 10 cm quadrats were selected, with adjacent quadrats separated by at least 10 m. Five quadrat samples were pooled and homogenized to form one composite biological replicate, yielding three independent composite replicates per crust category (effective *n* = 3). The fifteen quadrats were not treated as fifteen analytical replicates.

Before soil collection, the entire visible moss-crust layer was removed. Mineral soil from the upper 0–2 cm immediately beneath the base of the removed crust was then collected using sterile tools. Moss tissues were not included in the metagenomic or physicochemical analyses. Samples were transported on dry ice and stored at −80 °C until DNA extraction. Mixed patches were selected when the two mosses appeared intermingled and each occupied approximately half of the visible surface cover; the proportions were visually estimated and were not validated by image analysis or dry biomass.

### 2.2. Soil Physicochemical Analyses

Soil water content (SWC) was determined gravimetrically by drying fresh soil at 105 °C to constant mass. Air-dried soil was homogenized and passed through a 2 mm sieve before the remaining analyses. Soil pH was measured in a suspension of 10 g soil and 25 mL CO_2_-free water after 30 min of equilibration using a DZS-708L multiparameter analyzer (Shanghai INESA Scientific Instrument Co., Ltd., Shanghai, China). Total carbon (TC) and total nitrogen (TN) were measured by dry combustion with an elemental analyzer, soil organic matter (SOM) by potassium dichromate oxidation with external heating, and particle-size fractions by laser diffraction after organic-matter removal and sodium hexametaphosphate dispersion. Sand, silt, and clay were defined as 2–0.05, 0.05–0.002, and <0.002 mm, respectively, following standard soil-analysis procedures [19].

### 2.3. DNA Extraction, Library Preparation, and Read Quality Control

Total genomic DNA was extracted from 0.25 g of each composite below-crust soil sample using the DNeasy PowerSoil Pro Kit (Qiagen, Hilden, Germany). DNA integrity was checked by 1% agarose gel electrophoresis, and concentration and purity were assessed using a NanoDrop One spectrophotometer (Thermo Fisher Scientific, Wilmington, DE, USA) and Qubit 4 fluorometer (Thermo Fisher Scientific, Waltham, MA, USA), respectively. Extracts with a concentration ≥10 ng μL^−1^ passed the quality-control criterion; exact total extraction yields were not recorded. An equal input of 100 ng DNA per sample was used for library preparation.

Libraries were prepared using the NEBNext Ultra II DNA Library Prep Kit (New England Biolabs, Inc., Ipswich, MA, USA) for Illumina and sequenced on an Illumina NovaSeq 6000 (Illumina, Inc., San Diego, CA, USA) platform with 2 × 150 bp paired-end reads. Raw reads were processed with fastp v0.23.2 [20]. Bases with Phred scores < 15 were classified as unqualified; reads were discarded when >40% of their bases were unqualified. Adapters and reads with excessive ambiguous bases were removed, and reads shorter than 50 bp after trimming were excluded. Sample-level raw- and clean-read counts and Q30 outcomes are reported in Supplementary Table S3.

### 2.4. Assembly, Gene Prediction, and Abundance Normalization

Clean reads were assembled independently by sample using MEGAHIT v1.2.9 with the iterative *k*-mer series 21, 29, 39, 59, 79, 99, 119, and 141 [21]. Contigs < 500 bp were excluded. Open reading frames were predicted with Prodigal v2.6.3 in metagenomic mode (*-p meta*) [22]. No dedicated reference-based chimera assessment was performed. Assembly size, contigs > 500 bp, N50, and predicted ORF counts are reported by sample in Table S3.

**Figure 2:**
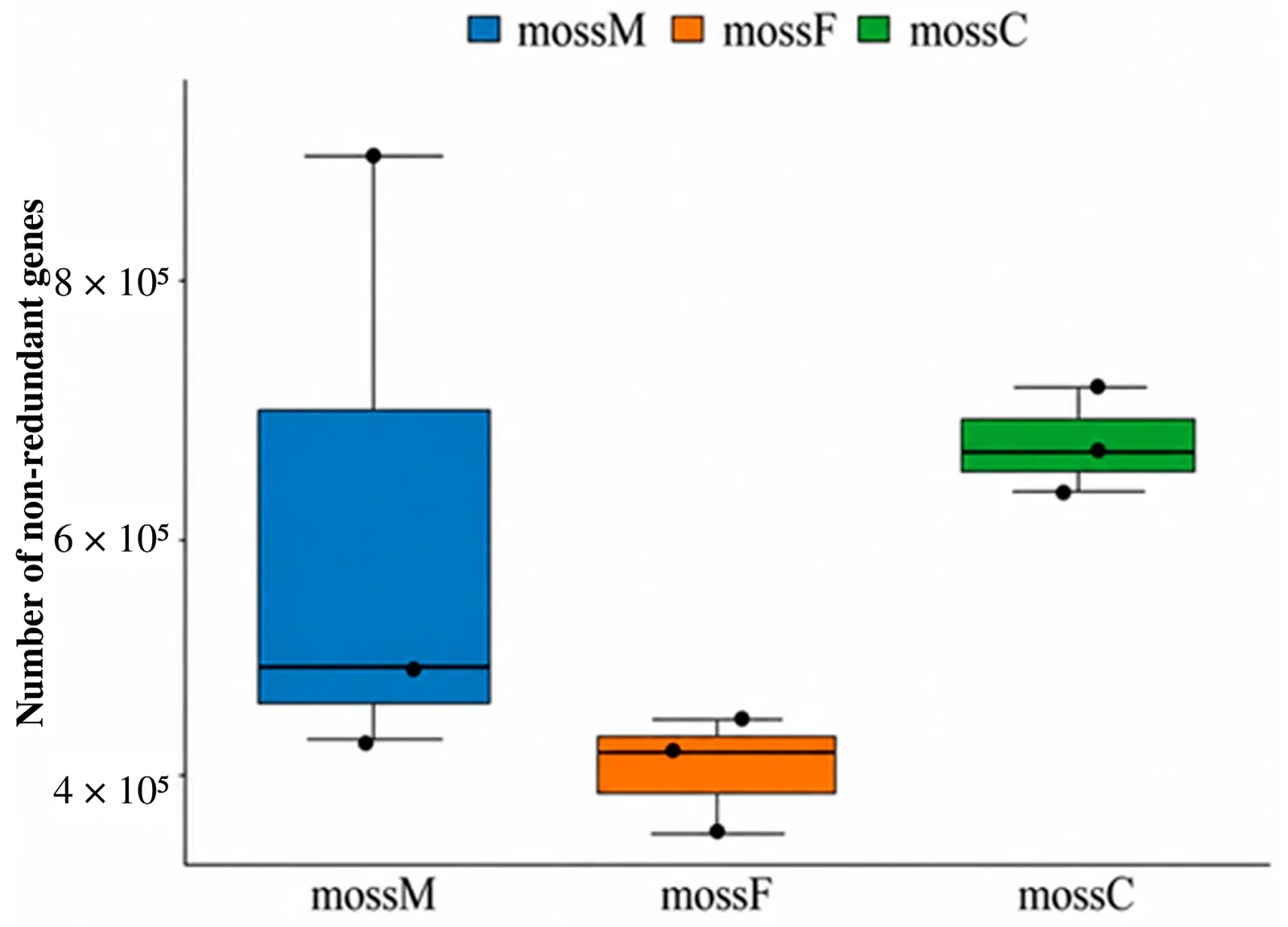
Number of non-redundant genes recovered from each independently assembled composite metagenome associated with mossM, mossF, and mossC. Boxes represent interquartile ranges, horizontal lines indicate medians, whiskers show observed ranges, and points are independent composite biological replicates (*n* = 3 per category). No inferential group comparison was performed.

Predicted protein-coding sequences were pooled and clustered with CD-HIT v4.8.1 at 95% amino-acid identity and 90% alignment coverage of the shorter sequence [23]. The longest sequence in each cluster was retained. The number displayed in Figure 2 is the number of non-redundant genes recovered from each independently assembled composite metagenome; it is not summed TPM, total mapped reads, or rarefaction-standardized gene richness. Clean reads were mapped to the catalog with Bowtie2 v2.4.5 [24], and gene-level relative representation was normalized as transcripts per million (TPM).

### 2.5. Taxonomic and Functional Annotation

Taxonomic profiles were generated using Kraken2 v2.1.2 against NCBI RefSeq release 220 [25]. Representative proteins were searched with DIAMOND BLASTP v2.1.6 against NCBI-NR using an *E*-value threshold of 1 × 10^−5^ [26]. KEGG Orthology identifiers were assigned with KofamScan v1.3.0 against the KOfam/KEGG database downloaded on 10 April 2023 [27].

Carbon-cycling genes were identified from KEGG annotations using a curated list of genes involved in carbon fixation, organic-carbon degradation, carbon oxidoreductase activity, and hemicellulose debranching; the full list, KO identifiers, pathways, and inclusion criteria are provided in Table S4. Nitrogen-cycling genes were identified by DIAMOND searches against NCycDB v20220430 at *E* < 1 × 10^−5^, sequence identity ≥40%, and alignment coverage ≥50%, and were cross-checked against KEGG nitrogen metabolism annotations.

Carbohydrate-active enzymes were annotated using HMMER against dbCAN HMMdb version 11, DIAMOND against CAZyDB release 08062022, and HMMER against the dbCAN-sub HMM database released in June 2022 [28]. Matches against dbCAN HMMdb and dbCAN-sub were retained at *E* < 1 × 10^−15^ and HMM coverage > 0.35. DIAMOND matches against CAZyDB were accepted at *E* < 1 × 10^−102^. Per-component and post-consensus counts are provided in Table S3.

### 2.6. Statistical Analyses

**Figure 3:**
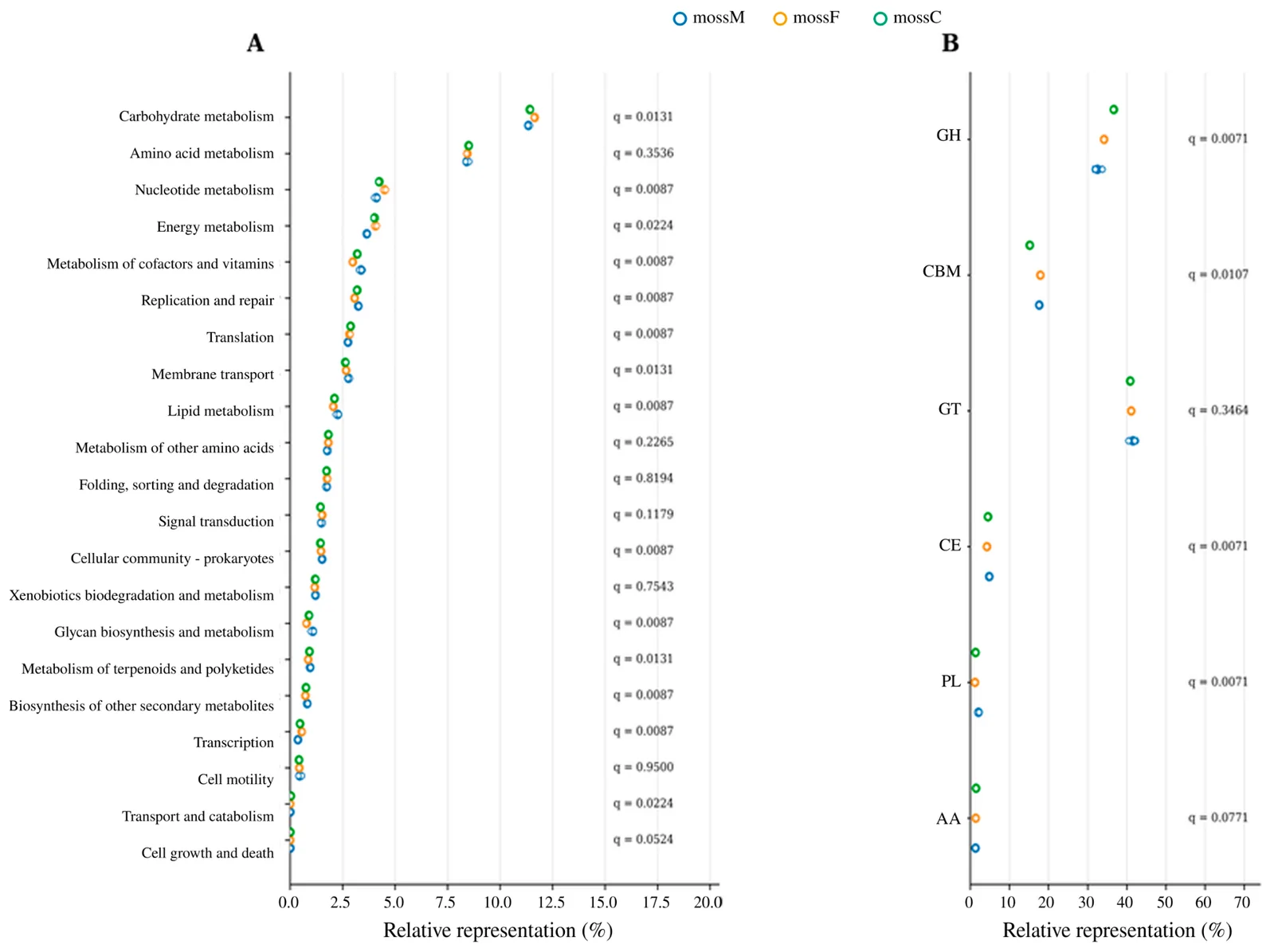
Relative functional profiles across moss biocrust categories. (**A**) Twenty-one specific KEGG Level 2 categories and (**B**) all six CAZyme classes. Filled circles and horizontal lines show group means ± SD; open circles show the three independent composite biological replicates. Omnibus *p* values were obtained from exact permutation tests of the one-way F statistic and adjusted separately across all 22 detected KEGG categories and all six CAZyme classes; BH-adjusted *p* values are shown to the right. The broad Global and overview maps category was included in the 22-test correction family and Table S2 but omitted from panel A for scale. GH, glycoside hydrolases; GT, glycosyltransferases; CBM, carbohydrate-binding modules; CE, carbohydrate esterases; PL, polysaccharide lyases; AA, auxiliary activities. Replicate-level values, raw *p* values, adjusted *p* values, and pairwise results are provided in Table S2.

**Figure 4:**
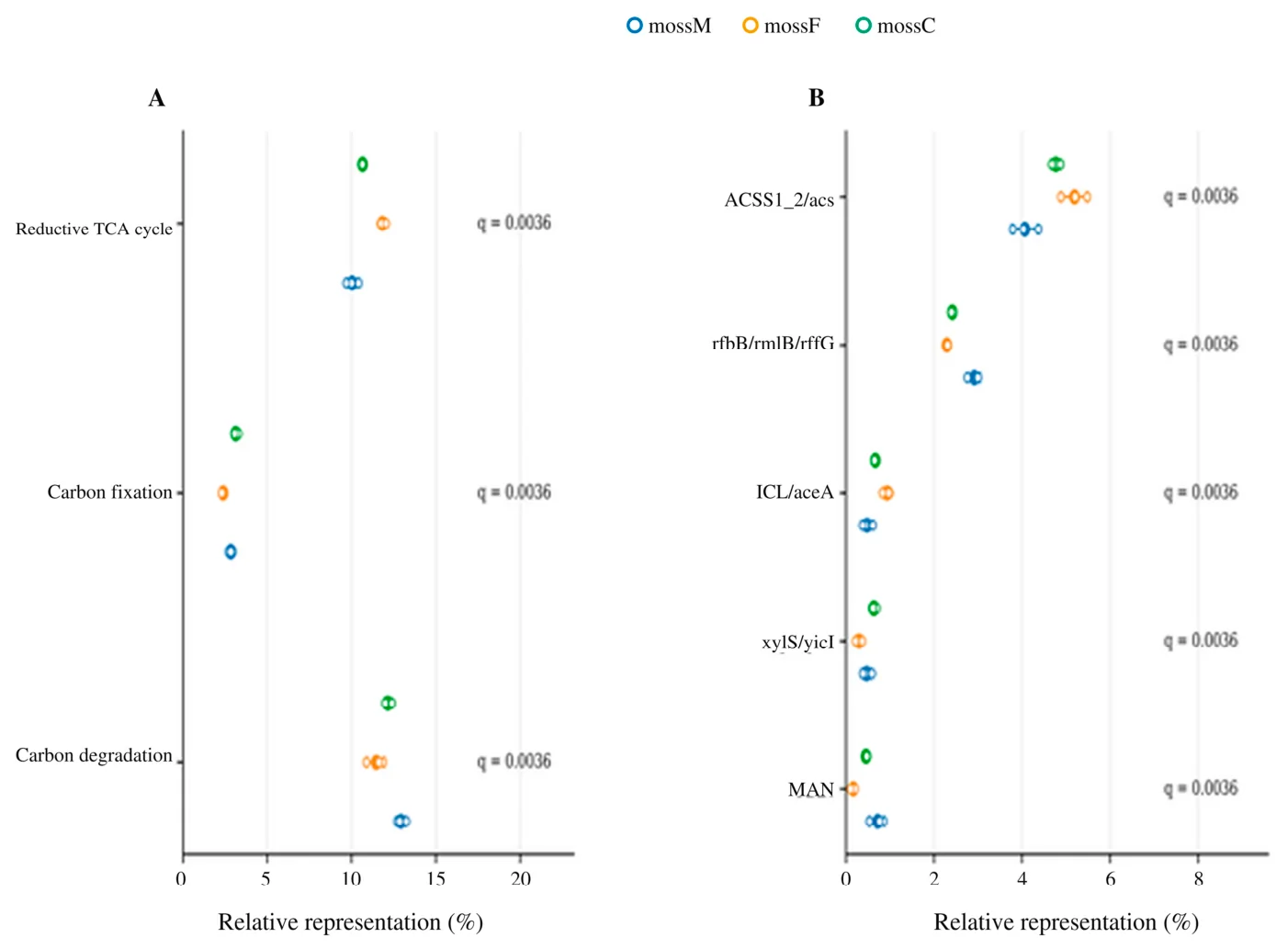
Carbon-cycling functional profiles across moss biocrust categories. Relative representation of (**A**) three carbon-cycling pathways and (**B**) five selected carbon-associated genes for which complete nine-sample source data were available. Filled circles and horizontal lines show group means ± SD; open circles show the three independent composite biological replicates. Omnibus *p* values were obtained from exact permutation tests of the one-way F statistic and adjusted separately within the three-pathway and five-gene families; BH-adjusted *p* values are shown to the right. Replicate-level values, raw *p* values, adjusted *p* values, and pairwise results are provided in Table S2.

**Figure 5:**
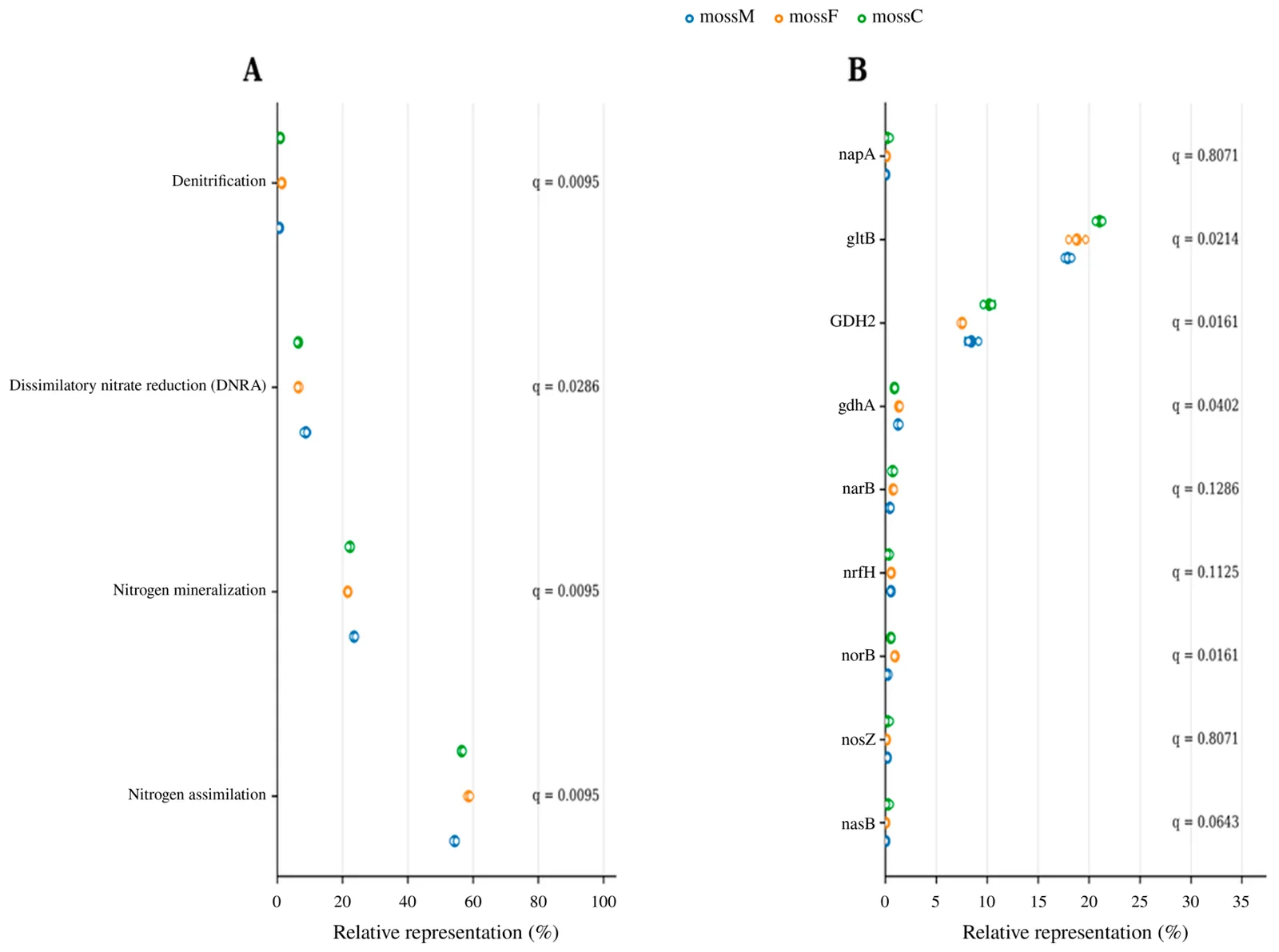
Nitrogen-cycling functional profiles across moss biocrust categories. Relative representation of (**A**) four nitrogen-cycling pathways and (**B**) nine selected nitrogen-associated genes. Filled circles and horizontal lines show group means ± SD; open circles show the three independent composite biological replicates. Omnibus *p* values were obtained from exact permutation tests of the one-way F statistic and adjusted separately within the four-pathway and nine-gene families; BH-adjusted *p* values are shown to the right. DNRA, dissimilatory nitrate reduction to ammonium; gltB, glutamate synthase large subunit; GDH2 and gdhA, glutamate dehydrogenase annotations; narB and nasB, nitrate-reductase annotations; nrfH, nitrite-reductase electron-transfer subunit; norB, nitric oxide reductase; nosZ, nitrous oxide reductase; napA, periplasmic nitrate reductase. Replicate-level values, raw *p* values, adjusted *p* values, and pairwise results are provided in Table S2.

Analyses were conducted in R v4.2.3. The independent unit was the composite biological replicate (*n* = 3 per crust category; nine samples total). Among-category comparisons of the eight soil properties and the functional features in Figure 3, Figure 4 and Figure 5 used exact permutation tests based on the one-way F statistic. For each variable, all 1680 allocations of nine samples to three labeled groups of size three were enumerated. Exploratory pairwise comparisons used exact two-sided permutation tests based on the absolute difference in group means, with all 20 allocations of six samples to two groups of size three enumerated. Replicate-level values, group means and SDs, test statistics, raw *p* values, and adjusted *p* values are provided in Tables S1 and S2.

Benjamini–Hochberg correction [29] was applied separately to seven prespecified omnibus families: eight soil properties; 22 KEGG Level 2 categories; six CAZyme classes; three carbon-cycling pathways; five selected carbon-associated genes; four nitrogen-cycling pathways; and nine selected nitrogen-associated genes. All *p* values in each family were retained irrespective of unadjusted significance. Soil-property pairwise *p* values were corrected together across all 24 variable-by-contrast tests. Pairwise *p* values for the functional features were corrected separately within each panel across all variable-by-contrast tests. The 128 predefined functional-feature–soil correlations, the three single-variable RDA tests, and the four taxonomic–functional Mantel tests each formed separate correction families. DESeq2 was not used. Complete Mantel-test statistics are provided in Supplementary Table S7.

Exploratory redundancy analysis (RDA) was conducted with vegan v2.6-4 [30], using 16 prespecified functional variables and standardized pH, TC, and SWC. No forward, stepwise, or VIF-driven selection was used. The full model contained all three variables, and robustness was evaluated with prespecified pH-only, TC-only, and SWC-only models. Adjusted R^2^ was calculated, and all 9! = 362,880 unrestricted permutations were enumerated. Spearman correlations between functional features and eight soil variables were likewise treated as exploratory associations.

For taxonomic–functional concordance, genus-level taxonomic profiles and KEGG pathway, CAZy family, carbon-cycling gene, and nitrogen-cycling gene profiles were converted to sample-wise relative abundances and square-root transformed. Bray–Curtis dissimilarity matrices were calculated, and Spearman Mantel tests compared the genus-level matrix with each functional matrix [31]. All 362,880 sample-label permutations were enumerated. These matrix-level tests do not identify the taxa carrying individual genes.

### 2.7. Calculation of Mixed-Crust Midpoint Deviations

For each functional category or gene, an approximate unweighted midpoint reference (*E*) was calculated from the two monospecific group means:

(1) $$E=\left({\bar{X}}_{mossC}+{\bar{X}}_{mossF}\right)/2$$

The percentage deviation of the mossM group mean from this reference was then calculated as:

(2) $$D(\%)=[({\bar{X}}_{mossM}-E)/E]\times 100$$

Positive and negative values indicate that the mossM mean was respectively above or below the reference. The deviation was calculated from the three group means only. Pairwise mossC × mossF combinations were not treated as independent expectations because they would reuse the same biological replicates.

## 3. Results

### 3.1. Soil Physicochemical Properties

Soil physicochemical properties differed overall among the three moss biocrust categories (Table 1). After Benjamini–Hochberg correction across the eight soil variables, exact one-factor permutation tests retained among-category differences in sand content (permutation *F* = 162.743, raw *p* = 0.0036, BH-adjusted *p* = 0.0095), silt content (F = 161.264, raw *p* = 0.0036, BH-adjusted *p* = 0.0095), clay content (F = 111.296, raw *p* = 0.0214, BH-adjusted *p* = 0.0286), soil organic matter (F = 44.411, raw *p* = 0.0214, BH-adjusted *p* = 0.0286), total carbon (F = 6.070, raw *p* = 0.0393, BH-adjusted *p* = 0.0449), total nitrogen (F = 41.115, raw *p* = 0.0143, BH-adjusted *p* = 0.0286), and soil water content (F = 100.396, raw *p* = 0.0036, BH-adjusted *p* = 0.0095). The overall difference in pH was not supported after correction (F = 3.937, raw *p* = BH-adjusted *p* = 0.0750).

At the group-mean level, mossC had the lowest sand content and the highest silt, clay, soil organic matter, total carbon, total nitrogen, and soil water content. MossF had the highest mean sand content, whereas mossM generally showed intermediate mean values. However, none of the 24 pairwise exact permutation comparisons remained supported after BH correction (all BH-adjusted *p* ≥ 0.1333). Therefore, these group-mean patterns are reported descriptively and are not interpreted as statistically supported differences between specific pairs of crust categories. The complete pairwise exact permutation results are provided in Supplementary Table S1.

### 3.2. Recovered Non-Redundant Genes

The number of non-redundant genes recovered from the independently assembled composite metagenomes varied among samples (Figure 2). At the group level, the median number of recovered genes followed the order mossF < mossM < mossC. MossF displayed the lowest median and a relatively narrow within-group range, whereas mossC displayed the highest median. MossM exhibited the widest within-group range, primarily because one composite sample contained substantially more recovered genes than the other two mossM samples. These assembly-derived gene counts are presented descriptively because no inferential group comparison was performed.

### 3.3. KEGG and CAZy Functional Profiles

All 22 detected KEGG Level 2 categories were included in the exact among-category permutation tests and one BH correction family. Fourteen categories retained omnibus support at adjusted *p* < 0.05 (Figure 3A; Table S2). MossM had the highest group mean for lipid metabolism, metabolism of cofactors and vitamins, biosynthesis of other secondary metabolites, metabolism of terpenoids and polyketides, glycan biosynthesis and metabolism, membrane transport, cellular community–prokaryotes, and replication and repair. MossF had the highest mean for carbohydrate, energy, and nucleotide metabolism and transcription, whereas mossC had the highest mean for translation and transport and catabolism. The broad umbrella category Global and overview maps was retained in the 22-test family and Table S2 but omitted from Figure 3A because its scale compressed the specific categories. None of the exact pairwise comparisons for Figure 3, Figure 4 and Figure 5 remained supported after their panel-specific BH corrections; group orderings reported below therefore refer to observed means.

GH constituted a large fraction of the CAZyme profile and had the highest group mean in mossC, whereas CBM had the highest mean in mossF (Figure 3B). CE and PL had their highest means in mossM. Omnibus differences were retained for GH, CBM, CE, and PL (adjusted *p* = 0.0071–0.0107), but not for GT or AA. These values are TPM-derived relative compositions within the CAZyme-annotated gene set and do not represent absolute gene copies. Replicate-level source values and complete statistics are provided in Table S2.

### 3.4. Carbon-Cycling Functions and Selected Genes

All three retained carbon-cycling pathways showed omnibus differences after BH correction (adjusted *p* = 0.0036; Figure 4A). Carbon degradation had the highest group mean in mossM, carbon fixation in mossC, and the reductive tricarboxylic acid (rTCA) cycle in mossF. The five selected carbon-associated genes also retained omnibus support (adjusted *p* = 0.0036; Figure 4B). *ACSS1_2/acs*, which encodes acetyl-CoA synthetase, and *ICL*/*aceA* had their highest means in mossF; *rfbB*/*rmlB*/*rffG* and *MAN* had their highest means in mossM; and *xylS*/*yicI* had its highest mean in mossC. These TPM-normalized patterns describe functional potential and do not demonstrate pathway activity.

### 3.5. Nitrogen-Cycling Functions and Selected Genes

Nitrogen-assimilation genes dominated the retained nitrogen functional profile and had the highest group mean in mossF (Figure 5A). Nitrogen mineralization and dissimilatory nitrate reduction to ammonium (DNRA) had their highest means in mossM, whereas denitrification had the lowest overall representation and its highest group mean in mossF. All four pathway-level omnibus tests remained supported after BH correction (adjusted *p* = 0.0095–0.0286). Among the nine selected genes (Figure 5B), *gltB* and *GDH2* had the highest means in mossC, while *gdhA* and *norB* had the highest means in mossF; these four genes retained omnibus support (adjusted *p* = 0.0161–0.0402). *nasB*, *narB*, *nrfH*, *nosZ*, and *napA* did not retain support after correction. Low-representation genes are interpreted cautiously, and complete replicate-level values are reported in Table S2.

### 3.6. Environmental Associations and Taxonomic–Functional Concordance

**Figure 6:**
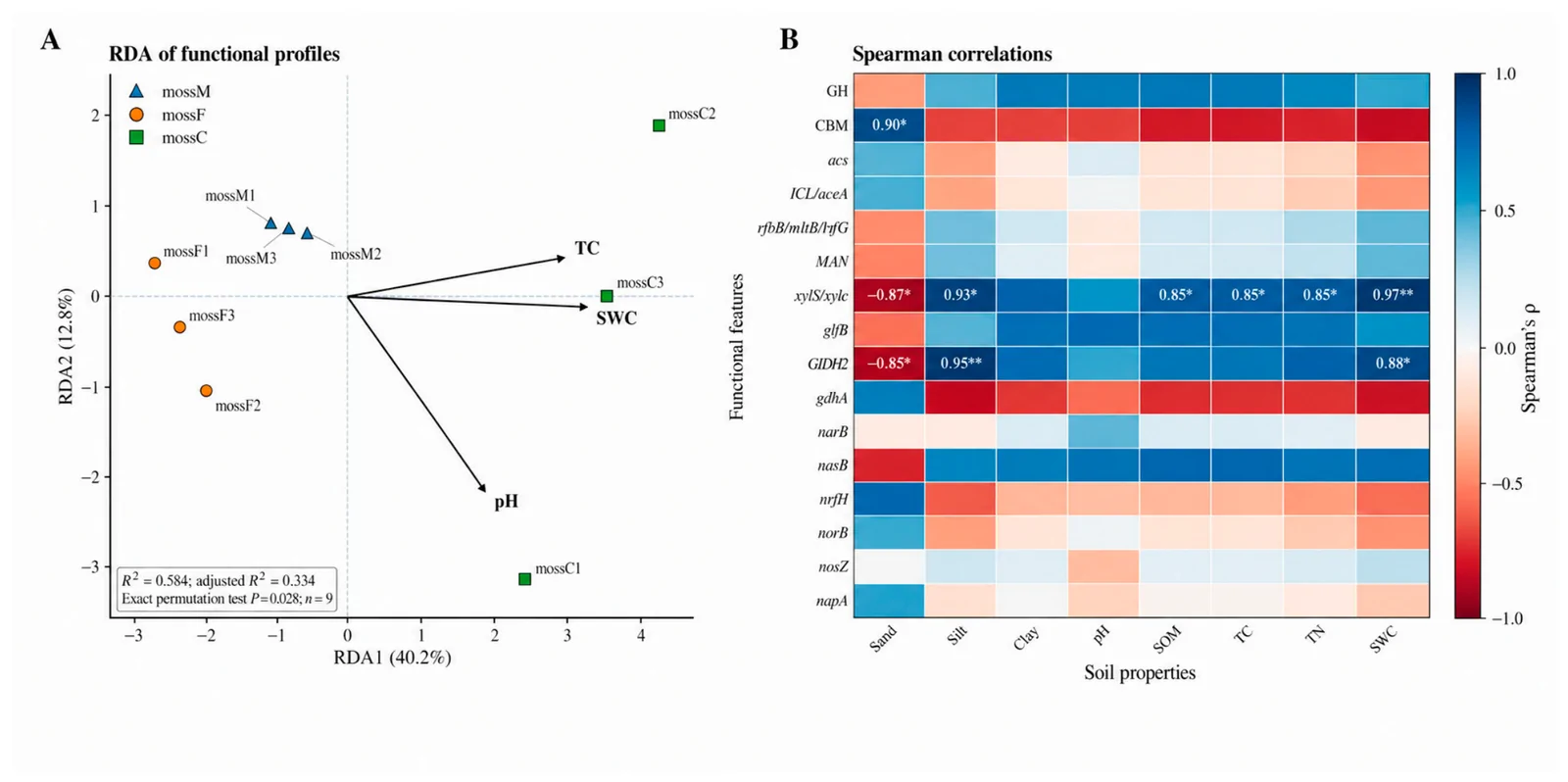
Associations between microbial functional profiles and soil physicochemical properties. (**A**) Exploratory RDA of nine composite biological samples. The full model included pH, TC, and SWC (R^2^ = 0.584, adjusted R^2^ = 0.334, constrained df = 3, residual df = 5, pseudo-*F* = 2.336, exact permutation *p* = 0.028340). The ordination shows associations and does not demonstrate independent environmental effects. (**B**) Spearman correlation heatmap. Blue represents positive correlations and red negative correlations. Spearman’s ρ is printed in the 10 cells that remained supported after Benjamini–Hochberg correction across all 128 correlations; * and ** indicate Benjamini–Hochberg-adjusted *q* values of <0.05 and <0.01, respectively. Complete coefficients and raw and adjusted *p* values are provided in Table S5.

The full RDA was based on nine composite samples, with 3 constrained and 5 residual degrees of freedom (Figure 6A). It explained 58.4% of the functional variation (R^2^ = 0.584), whereas the adjusted proportion was 33.4% (adjusted R^2^ = 0.334; pseudo-*F* = 2.336; exact permutation *p* = 0.028340). The pH-only model was not supported after correction (R^2^ = 0.235, adjusted R^2^ = 0.125, pseudo-*F* = 2.146, FDR-adjusted *p* = 0.084518). TC-only (R^2^ = 0.338, adjusted R^2^ = 0.244, pseudo-*F* = 3.575, FDR-adjusted *p* = 0.004386) and SWC-only models (R^2^ = 0.401, adjusted R^2^ = 0.316, pseudo-*F* = 4.692, FDR-adjusted *p* = 0.001190) retained supported associations. Complete results are reported in Table S6.

After one Benjamini–Hochberg correction across all 128 correlations, 10 associations remained supported (Figure 6B). CBM was positively associated with sand. *xylS*/*yicI* was negatively associated with sand and positively associated with silt, SOM, TC, TN, and SWC; *GDH2* was negatively associated with sand and positively associated with silt and SWC. Coefficients and raw and adjusted *p* values for all correlations are provided in Table S5.

Genus-level taxonomic dissimilarity was positively correlated with KEGG pathway dissimilarity (Mantel *r* = 0.571943, raw *p* = 0.007562, FDR-adjusted *p* = 0.008964), CAZy family dissimilarity (*r* = 0.682368, raw *p* = 0.000725, FDR-adjusted *p* = 0.002899), carbon-cycling gene dissimilarity (*r* = 0.632175, raw *p* = 0.002571, FDR-adjusted *p* = 0.005142), and nitrogen-cycling gene dissimilarity (*r* = 0.444015, raw *p* = 0.008964, FDR-adjusted *p* = 0.008964). These associations show concordant matrix-level variation but do not identify gene-carrying taxa or establish causation. Complete results are provided in Supplementary Table S7.

### 3.7. Descriptive Mixed-Crust Midpoint Deviations

**Figure 7:**
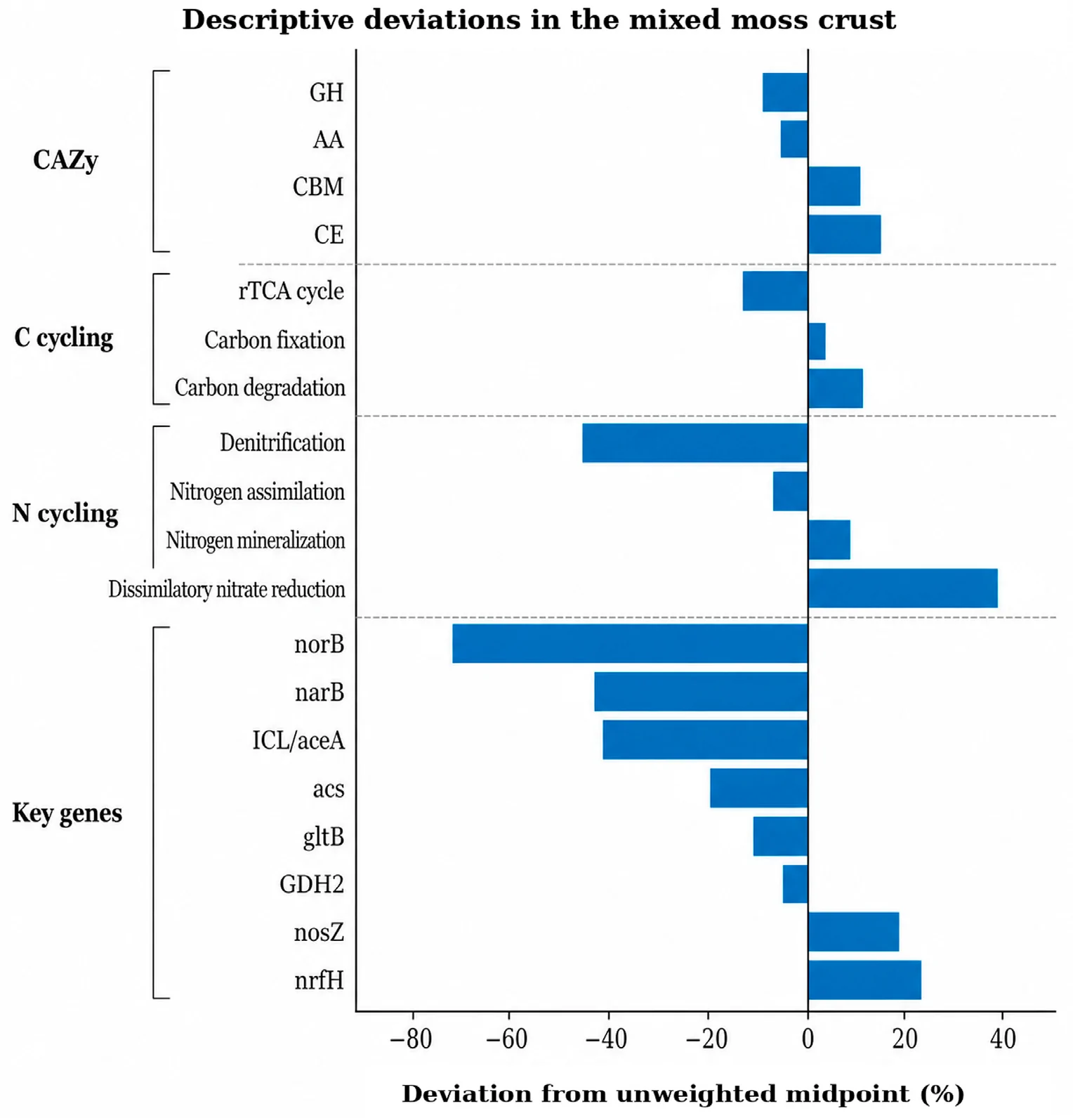
Descriptive deviations of microbial functional potential in mixed moss crusts from the approximate unweighted midpoint of the two monospecific crust categories. Horizontal dashed lines separate the CAZy, carbon cycling, nitrogen cycling, and key-gene groups. Each group mean was based on three independent composite biological replicates. No inferential test, confidence interval, FDR-adjusted *p* value, or significance star is shown. Because moss proportions were visually estimated, the reference is approximate and the values are interpreted only as exploratory descriptive deviations.

The mossM group mean showed both positive and negative deviations from the approximate unweighted midpoint reference (Figure 7). CBM, CE, carbon degradation, nitrogen mineralization, dissimilatory nitrate reduction, *nosZ*, and *nrfH* showed positive deviations, whereas GH, AA, the rTCA cycle, nitrogen assimilation, denitrification, *acs*, *ICL*/*aceA*, *gltB*, *GDH2*, *narB*, and *norB* showed negative deviations. Among the displayed variables, *norB* had the largest negative deviation: the mossM group mean was more than 60% below the monospecific midpoint. These values are descriptive and are not statistically confirmed biological non-additivity.

## 4. Discussion

### 4.1. Functional Differentiation Is Associated with Crust Type and Edaphic Conditions

The principal result is that moss crust category, accompanying edaphic conditions, and microbial functional gene profiles co-varied at the investigated site. MossC occurred in finer, wetter, more carbon- and nitrogen-rich soil and had greater relative representation of several carbohydrate-processing and nitrogen-assimilation genes. MossF and mossM occurred in coarser, comparatively nutrient-poor soil and showed different profiles. Biocrust succession and composition commonly covary with soil resources and microbial community structure [32,33,34,35,36]. The present design cannot determine whether soil conditions influenced moss establishment, moss development modified the soil, or reciprocal feedbacks generated both patterns.

The RDA and gene–soil correlations provided a more specific link between functional profiles and soil moisture, with the relative abundances of *xylS*/*yicI* and *GDH2* positively correlated with SWC. Alpine moss crusts undergo repeated desiccation–rewetting cycles, during which changes in water-film connectivity affect solute diffusion, substrate accessibility, and microbial metabolism [5,7,11]. During drying, disconnected water films restrict the movement of soluble substrates, while the concentration of dissolved salts and metabolites lowers external water potential and increases osmotic stress. Rewetting restores water-film connectivity and facilitates the transport of soluble carbon and nitrogen compounds through the crust–soil interface [5,7,11]. Within this moisture regime, the positive association of *xylS*/*yicI* with SWC is physiologically compatible with improved access to carbohydrate substrates under hydrated conditions, whereas the association of *GDH2* with SWC is compatible with moisture-dependent glutamate and ammonium metabolism [16]. However, neither gene is a specific marker of osmotic-stress tolerance, and SWC covaried with soil texture, SOM, TC, and TN [32,33,34,35,36]. These correlations should therefore be interpreted as moisture-associated patterns of functional potential rather than evidence that SWC or osmotic stress directly regulated gene expression or enzyme activity.

The wider range of recovered non-redundant gene counts in mossM was driven largely by one composite. The visually defined mixed patches may have differed in species proportions, shoot density, hydration, or trapped substrates, but sequencing depth, assembly performance, and community genomic complexity also influence gene recovery. Accordingly, this variability cannot be attributed solely to coexistence. Quantitative cover mapping and greater replicate-level sampling are needed to separate biological heterogeneity from technical variation.

### 4.2. Contrasting Carbon-Processing Potentials

The highest group means for GH and *xylS*/*yicI* occurred in mossC, consistent with greater genetic potential for hydrolytic processing of carbohydrates [37,38]. These patterns cannot be attributed specifically to *D. constrictus* litter quality because tissue chemistry was not measured. Higher SOM, TC, TN, finer texture, and SWC in mossC could also have affected substrate availability and microbial composition. Recent studies similarly show that biocrust state, hydration, and resource accumulation influence carbon cycling and microbial functional profiles [39,40,41].

MossF had the highest group means for *ACSS1_2/acs* and *ICL*/*aceA*, consistent with acetate activation and the glyoxylate shunt. Acetate and other C2 substrates could arise from fermentation products, microbial turnover, or soluble moss-derived carbon, but their concentrations and sources were not measured. MossM had the highest mean for carbon degradation and positive midpoint deviations for CBM and CE. These patterns could reflect a broader or more heterogeneous substrate spectrum, but TPM normalization means that greater relative representation of one feature can reduce the proportional representation of another without an absolute gene-copy change.

### 4.3. Nitrogen Acquisition, Retention, and Loss Potentials

The highest group mean for nitrogen-assimilation pathways occurred in mossF despite its lower measured TN. This is not necessarily contradictory because gene representation describes encoded acquisition potential rather than the size of the soil nitrogen pool, and nutrient limitation can favor investment in acquisition and assimilation functions. Conversely, *gltB* and *GDH2* had their highest means in mossC. Biocrust nitrogen transformations also vary with season, successional state, and hydration [4,32,42,43].

Dissimilatory-nitrate-reduction pathways had their highest group mean in mossM, consistent with genetic potential for a nitrogen-retention pathway because DNRA retains reactive nitrogen as ammonium [44]. Although mosses lack true roots, their rhizoids maintain close contact with the soil, and the C:N balance of moss-derived soluble compounds, senescent tissues, and rhizoid-associated detritus can influence the carbon and nitrogen supplied to the underlying mineral soil [5,11,12]. A relatively high supply of labile carbon compared with nitrate could favor nitrate reduction to ammonium, providing a possible stoichiometric context for the mossM pattern [44]. However, moss tissue and rhizoid-associated C:N ratios were not measured, so this explanation remains hypothetical. Nitrate availability, oxygen conditions, and DNRA rates were also not measured. Denitrification genes represented a small fraction of the profiles, although the pathway-level mean was highest in mossF. Coarse sandy soils are generally well aerated, which can restrict anaerobic denitrification; nevertheless, water retention by moss turfs and episodic wetting could temporarily reduce oxygen diffusion at the crust–soil interface and create transient low-oxygen microsites [5,40,43]. Because microsite oxygen dynamics were not measured, the occurrence of denitrification genes cannot be interpreted as evidence of active denitrification. The pronounced negative midpoint deviation of *norB* concerns TPM-normalized genetic potential for nitric oxide reduction and does not demonstrate lower expression, denitrification, or N_2_O production.

### 4.4. Interpretation of the Mixed-Crust Midpoint Deviations

The contrasting positive and negative deviations in mossM are more consistent with a redistribution of relative functional potential among categories than with uniform functional enhancement. Positive deviations in carbon degradation, CBM, CE, nitrogen mineralization, and DNRA contrasted with negative deviations in GH, AA, the rTCA cycle, nitrogen assimilation, and denitrification. Coexistence of the two moss species could increase microscale heterogeneity in soluble compounds, senescent tissues, rhizoid-associated detritus, trapped organic matter, moisture, and oxygen conditions [5,11,12]. This heterogeneous substrate environment could favor different decomposer and nitrogen-transforming guilds across adjacent microsites. Similarly, spatial variation in water retention and oxygen diffusion during repeated wetting–drying cycles could create hydrated, carbon-rich, and temporarily oxygen-limited microsites compatible with *DNRA*, alongside better-aerated microsites supporting aerobic decomposition [5,40,43,44]. However, the positive deviation of *nosZ* together with the negative deviations of norB and the overall denitrification pathway indicates that individual genes did not vary as a coordinated pathway. These gene-specific patterns could reflect taxonomic turnover, incomplete pathway distributions among taxa, or variation in low-abundance functional guilds [45,46,47], rather than corresponding changes in pathway activity. In addition, because TPM is a relative normalization measure, some deviations may represent compositional redistribution among annotated functions rather than absolute changes in gene abundance.

The arithmetic midpoint represents only an approximate additive expectation because it assumes equal and linear contributions from the two moss species. In this study, the approximately 1:1 mixture was determined visually from surface cover, whereas species biomass, shoot density, tissue chemistry, and rhizoid contributions were not quantified. Variation in species proportions, soil properties, and microbial community composition could therefore generate similar deviations. Moreover, the expected values derived from the single-species replicates were non-independent, and the small number of composite biological replicates limited estimation precision. The observed deviations should consequently be treated as exploratory, hypothesis-generating effect-size patterns rather than statistically confirmed non-additive effects caused by moss coexistence. Controlled mixture experiments combining quantified species proportions, matched soil conditions, absolute gene quantification, moisture and oxygen measurements, metatranscriptomics, and ^13^C/^15^N tracing are required to determine whether these patterns represent emergent changes in microbial activity and carbon or nitrogen transformation rates.

### 4.5. Limitations and Future Validation

Four limitations constrain inference. First, statistical analyses were based on three composite biological replicates per category; compositing increased spatial coverage within each analytical sample but prevented estimation of quadrat-level variance and limited power. Second, moss cover in mixed patches was visually estimated and biomass was not validated. Third, crust category covaried with texture, carbon, nitrogen, and water content, preventing separation of moss identity from associated edaphic conditions. Fourth, shotgun metagenomics measures functional potential; no transcriptomic, enzymatic, isotopic, or gas-flux measurements were available to verify activity.

Future work should preserve quadrat-level replication, quantify species cover and biomass, and use balanced mixture experiments that independently manipulate moss identity, mixture ratio, texture, and moisture. Species-specific tissue chemistry, litter C:N ratio, leachates, and rhizoid-associated microenvironments should be quantified. Metatranscriptomics, enzyme assays, qPCR/RT-qPCR, ^13^C and ^15^N stable-isotope tracing, soil respiration, and CO_2_, N_2_O, and CH_4_ flux measurements are required to test whether the observed gene profiles correspond to realized transformations. Broader seasonal and regional sampling is also needed because biocrust distributions and microbial responses vary across climates [10,48].

## 5. Conclusions

At the investigated alpine sandy site, below-crust microbial functional profiles differed among *Didymodon constrictus*, *D. ferrugineus*, and visually mixed moss crusts and covaried with accompanying soil conditions. MossC, mossF, and mossM showed contrasting relative representations of genes associated with carbohydrate processing, carbon metabolism, and nitrogen transformations. The mossM group mean showed positive and negative descriptive deviations from the approximate unweighted midpoint of the two monospecific group means, but these patterns are not statistically confirmed non-additive effects. The small number of independent composite replicates, visually estimated moss proportions, edaphic confounding, and absence of process-rate measurements limit causal inference and generalizability. Validation requires greater biological replication, quantitative mixture ratios, ^13^C/^15^N tracing, metatranscriptomics, enzyme assays, and greenhouse-gas flux measurements.

## Figures and Tables

**Table 1:** Soil physicochemical properties of the three moss biocrust types.

Variable	mossF	mossC	mossM	Permutation F	Raw *p*	BH-Adjusted *p*
Sand (%)	90.23 ± 0.29	82.75 ± 0.25	88.91 ± 0.38	162.743	0.0036	0.0095
Silt (%)	7.36 ± 0.04	11.90 ± 0.22	8.52 ± 0.23	161.264	0.0036	0.0095
Clay (%)	2.41 ± 0.05	5.35 ± 0.14	2.57 ± 0.23	111.296	0.0214	0.0286
pH	8.01 ± 0.13	8.39 ± 0.15	7.93 ± 0.06	3.937	0.0750	0.0750
SOM (g kg^−1^)	8.23 ± 0.32	12.26 ± 0.22	8.60 ± 0.43	44.411	0.0214	0.0286
Total C (g kg^−1^)	18.63 ± 1.06	23.73 ± 1.23	19.93 ± 0.92	6.070	0.0393	0.0449
Total N (g kg^−1^)	0.079 ± 0.003	0.142 ± 0.005	0.086 ± 0.007	41.115	0.0143	0.0286
Soil water content (%)	14.25 ± 0.21	18.49 ± 0.28	15.34 ± 0.14	100.396	0.0036	0.0095

Values are means ± SE (*n* = 3 composite biological replicates per category). Omnibus differences were evaluated using exact one-factor permutation F tests (all 1680 allocations), with Benjamini–Hochberg correction across the eight soil variables. The full pairwise results are provided in Supplementary Table S1.

## Data Availability

The raw metagenomic sequencing reads have been submitted to the NCBI Sequence Read Archive under BioProject accession number PRJNA1456289 and will be publicly available upon publication.

## Supplementary Materials

The following supporting information can be downloaded at: https://www.mdpi.com/article/10.3390/biology15161372/s1, Table S1, replicate-level soil physicochemical data and complete statistics; Table S2, replicate-level source data and statistics for Figure 3, Figure 4 and Figure 5; Table S3, sequencing, assembly, mapping, annotation, and dbCAN component outcomes; Table S4, curated carbon-cycling gene list with KO and pathway assignments; Table S5, all 128 Spearman correlations with raw and adjusted *p* values; Table S6, full and single-variable RDA results; Table S7, taxonomic–functional Mantel-test results. Supplementary Data include sample metadata, raw mapped-read counts, TPM-normalized matrices, annotation outputs, and scripts used for statistical analysis and figure generation.

## Abbreviations

The following abbreviations are used in this manuscript:

mossC	Didymodon constrictus crusts
mossF	Didymodon ferrugineus crusts
mossM	mixed crusts co-dominated by both species
